# Cell assembly dynamics of sparse inhibitory networks: a simple model for the activity of the Medium Spiny Neurons

**DOI:** 10.1186/1471-2202-16-S1-P14

**Published:** 2015-12-18

**Authors:** David Angulo-Garcia, Alessandro Torcini, Joshua D Berke

**Affiliations:** 1 Istituto dei Sistemi Complessi, Consiglio Nazionale delle Ricerche (CNR), via Madonna del Piano 10, Sesto Fiorentino, Italy I-50019; 2 Department of Psychology, University of Michigan, Ann Arbor, 530 Church St., Ann Arbor, MI 48104, USA

## 

**Figure 1:**
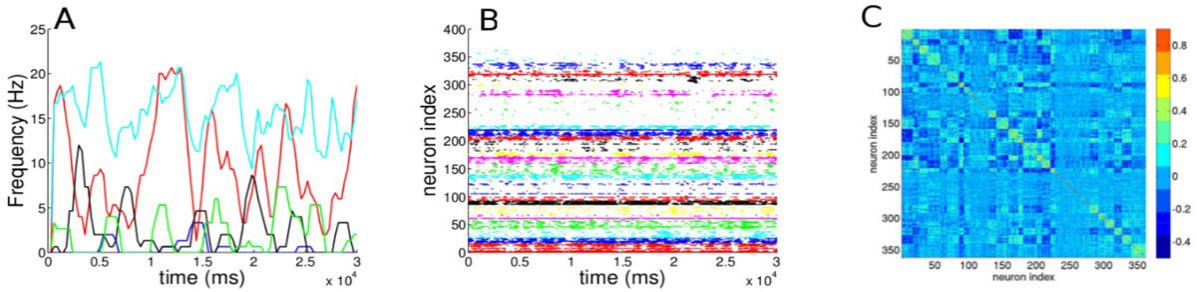
**A. Firing rates of 5 selected neurons**; **B**. raster plot activity, the firing times are colored accordingly to the assembly the neurons belong to; **C**. cross-correlation matrix of the firing rates. The neurons in panel B and C are clusterized accordingly to the correlation of their firing rates, by employing the k-means algorithm.

Here we show that a simple inhibitory network model, made of sparsely connected Leaky Integrate and Fire (LIF) neurons, is able to retrieve some of the relevant dynamical features of a striatal network, in particular the appearance of cell assembly dynamics as it has been reported in in-vitro experiments of rats striatum [1]. In a first approach, we discuss how our simple model is consistent with the findings in [2,3]. For an optimal choice of the model parameters, the response of the neurons to uniformly distributed constant inputs, occurs in a bursting fashion. As shown in Figure. 1B, the neurons organize their dynamics in groups with correlated bursting-like activity, displaying typical recurrent patterns, similarly to the dynamics of Medium Spiny Neurons (MSNs). Furthermore, the firing of the neurons taking part in this "structured" cell assembly dynamics is characterized by a high variability, as shown in Figure 1A for a few representative neurons. This high variability is reflected in a coefficient of variation of the interspike-interval (ISI) larger than one. An important aspect of the dynamics of the MSNs is the emergence of coexisting correlated and anti-correlated assemblies, as reported in the experimental work by Carrillo-Reid et al. [1]. Indeed also in our system this aspect is present, as revealed by examining the cross-correlation matrix of the firing rates shown in Figure 1C. Here the neurons are grouped in assemblies accordingly to their level of correlation (as in Figure 1B) and it is evident that the correlated activities within the neuronal assemblies can be highly anti-correlated with other cells in the network.
